# Transcript and protein profiling identify candidate gene sets of potential adaptive significance in New Zealand *Pachycladon*

**DOI:** 10.1186/1471-2148-10-151

**Published:** 2010-05-20

**Authors:** Claudia Voelckel, Mehdi Mirzaei, Michael Reichelt, Zhiwei Luo, Dana Pascovici, Peter B Heenan, Silvia Schmidt, Bart Janssen, Paul A Haynes, Peter J Lockhart

**Affiliations:** 1 Allan Wilson Centre for Molecular Ecology and Evolution, Massey University, Palmerston North, New Zealand; 2 Department of Chemistry and Biomolecular Sciences, Macquarie University, Sydney, Australia; 3 Max Planck Institute for Chemical Ecology, Jena, Germany; 4 Plant and Food Research, Mount Albert, Auckland, New Zealand; 5 Australian Proteome Analysis Facility, Macquarie University, Sydney, Australia; 6 Landcare Research, Lincoln, New Zealand

## Abstract

**Background:**

Transcript profiling of closely related species provides a means for identifying genes potentially important in species diversification. However, the predictive value of transcript profiling for inferring downstream-physiological processes has been unclear. In the present study we use shotgun proteomics to validate inferences from microarray studies regarding physiological differences in three *Pachycladon *species. We compare transcript and protein profiling and evaluate their predictive value for inferring glucosinolate chemotypes characteristic of these species.

**Results:**

Evidence from heterologous microarrays and shotgun proteomics revealed differential expression of genes involved in glucosinolate hydrolysis (myrosinase-associated proteins) and biosynthesis (methylthioalkylmalate isomerase and dehydrogenase), the interconversion of carbon dioxide and bicarbonate (carbonic anhydrases), water use efficiency (ascorbate peroxidase, 2 cys peroxiredoxin, 20 kDa chloroplastic chaperonin, mitochondrial succinyl CoA ligase) and others (glutathione-S-transferase, serine racemase, vegetative storage proteins, genes related to translation and photosynthesis). Differences in glucosinolate hydrolysis products were directly confirmed. Overall, prediction of protein abundances from transcript profiles was stronger than prediction of transcript abundance from protein profiles. Protein profiles also proved to be more accurate predictors of glucosinolate profiles than transcript profiles. The similarity of species profiles for both transcripts and proteins reflected previously inferred phylogenetic relationships while glucosinolate chemotypes did not.

**Conclusions:**

We have used transcript and protein profiling to predict physiological processes that evolved differently during diversification of three *Pachycladon *species. This approach has also identified candidate genes potentially important in adaptation, which are now the focus of ongoing study. Our results indicate that protein profiling provides a valuable tool for validating transcript profiles in studies of adaptive divergence.

## Background

It has been postulated that genes of co-ordinated function that offer an organism some selective advantage (adaptive gene sets) might be identifiable in studies that examine differential gene expression (DGE) across species ranges and heterogeneous environments [1,2]. Similarly, DGE studies that compare expression profiles of closely related species, such as those that have formed during Late Tertiary radiation of New Zealand's alpine genera, might also help to identify candidate genes that have played a role in species diversification. To investigate this approach, in an earlier study we compared microarray expression profiles for natural populations of two closely related alpine plant species from the largely endemic New Zealand genus *Pachycladon *(Brassicaceae). Analyses of logfold ratios and ontology analyses in this experiment suggested that secondary metabolite profiles differed among and between populations of these two species. HPLC-UV/GC-MS analyses confirmed these predictions, leading to the hypothesis that herbivory was a major driver of diversification of *P. fastigiatum *and *P. enysii *[3]. These results provided encouragement that transcriptome profiling used in a predictive sense provided a promising approach for helping to identify putative adaptations between closely related species.

However, a potential shortcoming with this approach is that gene expression differences between species may also result from genetic drift [4]. Moreover, due to posttranslational modifications, transcript abundances may not correlate with protein abundances [5]. Profiling of proteomes, which have different underlying evolutionary dynamics from transcriptomes [6], provides an alternative approach for the prediction of downstream physiological processes. In the present study we have compared findings from gene expression profiles obtained using heterologous microarrays [3] with findings from protein expression profiles generated by shotgun proteomics. During shotgun proteomics, proteins levels are calculated by determining the number of spectral counts that identify a protein [7] and proteins are identified by searching MS-MS spectra of peptides against reference protein data bases. Our aim was to determine to what extent gene expression profiles were indicative of protein expression profiles and vice versa, and to evaluate both methods with respect to their potential for predicting physiological differences among New Zealand *Pachycladon *species. For this purpose, we also characterized glucosinolate chemotypes and evaluated the extent to which these were predicted from the protein and transcript expression data.

The genus *Pachycladon *is of allopolyploid origin (< 2 Mya:[8]) and has diversified into ten morphologically and ecologically diverse species within the last million years. Nine of the species are endemic to the Southern Alps of New Zealand and one is native to Tasmania. The nine New Zealand species form three distinct groups distinguished by their genetic characteristics, morphological features and habitat preferences [9]. Species differ markedly in their preference for certain soil types. For example, the group consisting of *P. novae-zelandiae *and *P. wallii *are restricted to soils derived from schist whereas another group consisting of *P. fastigiatum*, *P. enysii* and *P. stellatum *are confined to greywacke soils. There are also the limestone specialist *P. fasciarium *and the basicole *P. exile*. The remaining two species, *P. cheesemanii *and *P. latisiliquum *are geological generalists. These preferences have led to the hypothesis that adaptation to different soils has to a large part driven diversification and evolution of distinct *Pachycladon *species. Since species also differ in their altitudinal ranges [10], adaptation to different altitudes has been suggested as another possible driver of diversification.

In this study, we compared three *Pachycladon *species with distinct ecological attributes. These species were *P. cheesemanii *(CH), *P. exile *(EX) and *P. novae-zelandiae *(NZ). The former two are sister species [9] and resemble close overseas *Pachycladon *relatives *Transberingia *and *Crucihimalaya *[11]. *P. cheesemanii *and *P. exile *are both polycarpic perennials with a woody caudex, exhibit leaf heterophylly with ovate to broadly elliptic early (basal) leaves with branched hairs on the lamina and petiole and serrate to lobed later (cauline) leaves; they have slender and terminal inflorescences and narrow petals and terete (cylindrical) siliques with uniseriate wingless seeds. *P. exile *is much smaller in all parts than *P. cheesemanii. P. cheesemanii *occurs along a wide altitudinal range (10-1600 m) on greywacke, semi-schist, schist and plutonics substrates, it is a true generalist. *P. exile *is a lowland species (25-500 m) that grows on base-rich soils. It was previously recorded from a few sites in Central Otago but is now known with certainty from only one limestone site and has recently been classified as nationally critical in New Zealand [12]. *P. novae-zelandiae *is also a polycarpic perennial with a semi-woody caudex but is morphologically distinct from *P. cheesemanii *and *P. exile*. It does not produce heteroblastic leaves. Rather it produces oblong to elliptic leaves that are either lobed or crenate and are either hairy (simple or branched trichomes) or glabrous; it has lateral inflorescences, and laterally compressed siliques with biseriate wingless seeds. *P. novae-zelandiae *grows at much higher altitudes (1080-2031 m) and is confined to schist soils. In summary, *P. cheesemanii *and *P. exile *are morphologically similar, lower altitude species that are very closely related but which differ in their soil substrate specificities (generalist and basicole respectively). In contrast, *P. novae-zelandiae *is a more distantly related alpine species restricted to schist. Given their different ecologies and degrees of relatedness, the three species are expected to differ in their gene and protein expression profiles. In the instances that protein profiles corroborate inferences from comparative transcript analyses, these observations have been used to predict physiological differences between species as well as candidate genes for adaptive diversification.

## Results

### Transcriptome - proteome correlations

To investigate the extent to which transcriptional patterns were congruent with protein expression patterns, we calculated correlation coefficients for 1074 genes surveyed by both transcript and protein profiling (table 1, A). Given the non-normal distributions of both the log2 spectral abundance factors and the log2 fluorescence intensities, we used Spearman's rank correlation coefficient. Each species' proteome correlated most strongly with its own transcriptome with the highest correlation observed in *P. cheesemanni *(0.52) and the lowest in *P. novae-zelandiae *(0.34). The transcript profile (for 1074 genes) for each species correlated most strongly with the proteome (for 1074 genes) of *P. cheesemanii*. This was largely due to the fact that the three transcriptomes correlated very strongly with one another (0.74-0.91, table 1, B) and the *P. cheesemanii *transcriptome had the highest correlation with its own proteome. The three proteomes correlated much more weakly with each other (0.59-0.75, table 1, C) than did the three transcriptomes, indicating greater differences between the protein profiles than between the transcript profiles. Correlations between both transcriptomes and proteomes mirrored phylogenetic relationships with the profiles of sister species *P. cheesemanii *and *P. exile *being most similar. Interestingly, both the transcriptome and proteome of *P. novae-zelandiae *were found to be more similar to those of *P. exile *than to those of *P. cheesemanii*.

**Table 1:** Correlations of transcript and protein expression statistics of a common gene set

A	CH_P	EX_P	NZ_P	B	CH_T	EX_T	NZ_T	C	CH_P	EX_P	NZ_P
CH_T	**0.52**	0.43	0.30	CH_T	1.00	0.91	0.74	CH_P	1.00	0.75	0.59
EX_T	**0.47**	**0.45**	0.32	EX_T		1.00	0.83	EX_P		1.00	0.72
NZ_T	**0.40**	0.36	**0.34**	NZ_T			1.00	NZ_P			1.00

Pairwise Spearman rank correlation coefficients between **A) **protein (P) and transcript (T) expression statistics (log2 normalized spectral abundance factors vs log2 fluroescense intensities), **B) **transcript expression statistics and **C) **protein expression statistics for 1074 genes surveyed by both microarrays and shotgun proteomics of three *Pachycladon *species. Species abbreviations: CH: *P. cheesemanii*, EX: *P. exile*, NZ: *P. novae-zelandiae*. The highest correlation coefficients for each species are depicted in bold. Note that the proteome of each species correlates most strongly with its own transcriptome (A). Furthermore, transcriptomes and proteomes of CH correlate more strongly with those of EX than with those of NZ (B, C), reflecting the closer phylogenetic relationship between CH and EX as opposed to CH and NZ.

### Overlap in transcript and protein up-regulation

**Figure 1:**
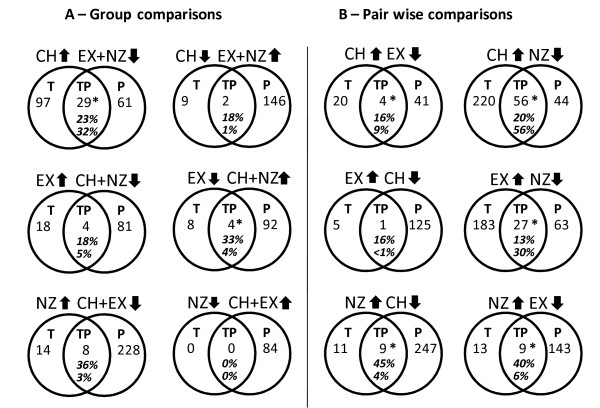
**Overlap in differential expression patterns from both transcript and protein profiling**. Numbers of differentially expressed genes in *P. cheesemanii *(CH), *P. exile *(EX) and *P. novae-zelandiae *(NZ) identified by transcript (T) profiling, protein profiling (P) or both (TP). Differential expression is defined as significantly higher or lower expression in one species compared with the other two combined (panel A - 6 group comparisons) or separately (panel B - 6 pair wise comparisons) and was determined by linear model analysis (transcripts) or Wilcoxon rank and t-tests (proteins). Numbers are corrected for relative sizes of transcript and protein data sets. In other words, only a subset of all differentially expressed transcripts and proteins is given. This subset consists of genes found amongst the 1074 loci surveyed by both transcript and protein profiling. For a summary of all differentially expressed transcripts and proteins refer to table 2. Upper percentages depict the proportion of transcripts confirmed by protein analysis and lower percentages depict the proportion of proteins confirmed by transcript analysis (see text for details). Statistically significant overlap (p value < 0.05%) is denoted with a star. For locus IDs, gene descriptions, transcript and protein expression statistics of genes identified as being up-regulated by both methods (TP) in CH, EX and NZ see table 3.

For each of the three *Pachycladon s*pecies, we identified up- and down-regulated transcripts and proteins using linear model analysis and Wilcoxon rank and t-tests, respectively. The number of up- and down-regulated transcripts and proteins are summarized in table 2 and gene identifiers, descriptions, transcript and protein expression statistics are provided in additional file 1, table S1. We compared expression in each species with expression in the other two species combined (6 group comparisons) and separately (6 pair wise comparisons). For example, expression in CH was compared to expression in EX+NZ (group comparison) but also to EX and NZ separately (pair wise comparisons). For each of the twelve comparisons, we intersected the lists of up-regulated transcripts with lists of up-regulated proteins and found significant overlap (i.e. different from random expectations given the size of our data sets) in seven cases (figure 1). The extent of overlap between transcript and protein profiles is limited partially as a result of relative sizes of the different data sets (transcript data set: 9404 genes, protein data set: 1489 genes, overlap of both data sets: 1074 genes). For example, of the 371 transcripts up-regulated in *P. cheesemanii *when compared with NZ+EX combined, only 126 were present in the protein data set. This means the 29 genes found up-regulated by both transcript and protein profiling in *P. cheesemanii *represent an overlap of 23% (29 of 126). Conversely, of the 124 proteins up-regulated in *P. cheesemanii *only 90 were present in the transcript data set. In this case we have found 32% (29 of 90) of the transcripts maximally identifiable by transcript profiling. Interestingly, across the three *Pachycladon *species, an average of 23.6% of the transcript patterns were confirmed by protein patterns (average of upper percentages in figure 1) whereas on average only 12.6% of the protein patterns were predicted by transcript patterns (average of lower percentages in figure 1). These percentages did differ greatly between species (figure 1). Transcript patterns were best confirmed by proteins patterns in *P. novae-zelandiae *(average of 30.2%) and protein patterns were best confirmed by transcript patterns in *P. cheesemanii *(average of 24.5%). The disagreement of transcript and protein profiling remaining after correcting for different numbers of genes captured by both analyses presumably is explained by the different kinetics of protein and transcript accumulation and degradation.

**Table 2:** Numbers of up-regulated transcripts and proteins in *P. cheesemanii*, *P. exile *and *P. novae-zelandiae*

	CH up	CH down	CH up	CH up
	EX+NZ down	EX+NZ up	EX down	NZ down
**T**	371	74	69	936
**P**	124	173	60	141

	**EX up**	**EX down**	**EX up**	**EX up**
	**CH+NZ down**	**CH+NZ up**	**CH down**	**NZ down**

**T**	134	70	45	946
**P**	109	136	161	110

	**NZ up**	**NZ down**	**NZ up**	**NZ up**
	**CH+EX down**	**CH+EX up**	**CH down**	**EX down**

**T**	305	1184	221	297
**P**	321	109	359	211

Expression of transcripts (T) and proteins (P) in each species (*P. cheesemanii*, CH, *P. exile*, EX, *P. novae-zelandiae*, NZ) was compared with expression in the remaining two species combined (6 group comparisons) and separately (6 pairwise comparisons). For example, 371 transcripts and 124 proteins were up-regulated in CH when compared with EX and NZ combined and 74 transcripts and 173 proteins were down-regulated in CH when compared with EX and NZ combined. Sixty nine transcripts and 60 proteins were up-regulated in CH when compared only to EX and 936 transcripts and 141 proteins were up-regulated in CH when compared only to NZ. The total numbers of transcripts and proteins investigated by microarray analysis and shotgun proteomics were 9601 and 1489, respectively. Locus IDs, gene descriptions, logarithmized expression ratios and adjusted p-values of differentially expressed transcripts and proteins for each of the 12 comparison are compiled in additional file 1, table S1.

Our goal was to identify genes specifically up- and down-regulated in each species with respect to the other two (group comparisons) in the set of 1074 genes that were covered by both transcript and protein profiling. In these comparisons the greatest overlap between transcript and protein profiles was found for *P. cheesemanii *(29 genes up-regulated, 2 genes down-regulated), followed by *P. exile *(4 genes up-regulated, 4 genes down-regulated) and *P. novae-zelandiae *(8 genes up-regulated, no overlap in down-regulation). Gene identifiers, descriptions, and expression statistics for these are compiled in table 3. From the genes equally identified by both approaches, some interesting observations and predictions can be derived. For example, three isoforms of carbonic anhydrases were differentially expressed in *P. cheesemanii *(At3g01500, AtβCA1, chloroplast-located, At5g14740, AtβCA2, cytosol-located) and *P. novae-zelandiae *(At1g23730, AtβCA3, cytosol-located) (table 3). These genes catalyze the interconversion of carbon dioxide and bicarbonate and have been shown to be involved in photosynthesis, respiration, pH regulation, inorganic carbon transport, ion transport, and water and electrolyte balance [13]. Other genes found up-regulated by both approaches in *P. cheesemanii *are genes involved in translation, photosynthesis light reactions, the calvin cycle, pentose phosphate pathway, fatty acid beta oxidation, serine metabolism, oxidative stress, protein folding and others (table 3). The four commonly up-regulated genes in *P. exile *are associated with very different processes such as Calvin cycle, S-adenosylmethione biosynthetic process, carbohydrate metabolism and others (table 3). In *P. novae-zelandiae*, two genes encoding vegetative storage proteins are up-regulated which have been implicated in responses to insects and other stress factors. Also up-regulated in *P. novae-zelandiae *are genes involved in transport, toxin catabolism and response to oxidative stress (table 3). Two and four genes were found to be down-regulated by transcript and protein profiling in *P. cheesemanii *and *P. exile*, respectively. The former two are involved in cell wall modification (pectinmethylesterase) and carbohydrate metabolism (glycosyl hydrolyase) and the latter four represent the two vegetative storage proteins up-regulated in *P. novae-zelandiae*, a universal stress protein and a phosphoglucomutase (table 3). Since none of the down-regulated transcripts in *P. novae-zelandiae *(table 2) were captured by the common set of 1074 genes, no overlap in down-regulated transcripts and proteins was observed in *P. novae-zelandiae*.

**Table 3:** Genes up-regulated in *P. cheesemanii*, *P. exile *and *P. novae-zelandiae *in transcript and protein profiling

		T	P			T	P
Locus	Description	LogFC	LogFC	Locus	Description	LogFC	LogFC
***P. cheesemanii *(29 genes up-regulated)**				***P. cheesemanii *(2 genes down-regulated)**			
***Translation***				***Cell wall modification***			
At1g05190	ribosomal protein L6	1.6	1.6	At1g11580	pectin methylesterase	-1.1	-1
At3g02560	40S ribosomal protein S7	1.0	2.9	***Carbohydrate metabolism***			
At5g13510	ribosomal protein L10 family protein	1.0	1.7	At4g19810	glycosyl hydrolase family 18 protein	-0.8	-1.4
At5g30510	30S ribosomal protein S1	1.5	1.9				
At5g54600	50S ribosomal protein L24	0.8	3.7	***P. exilis *(4 genes up-regulated)**			
***Photosynthesis light phase***				***Citrate cycle***			
At1g52230	photosystem I subunit H2	2.4	2.3	At2g20420	succinyl-CoA ligase (GDP-forming) beta	0.7	0.9
At4g21280	photosystem II subunit Q	1.2	1.8	***S-adenosylmethionine biosynthetic process***			
At4g03280	Rieske FeS center of cytochrome b6f	1.2	1.2	At2g36880	methionine adenosyl transferase 3	0.7	1.2
At5g64040	photosystem I subunit N	1.8	4.7	***Carbohydrate metabolism***			
At4g04640	chloroplast ATP synthase gamma subunit	1.3	0.8	At5g11720	alpha-glucosidase 1	0.7	5.0
At3g01480	chloroplast cyclophilin	1.3	2.0	***Other***			
***Calvin cycle***				At4g34180	cyclase family protein	0.9	1.6
At1g12900	gap dehydrogenase a subunit 2	1.7	0.6				
At1g32060	phosphoribulokinase (PRK)	1.5	0.6	***P. exilis *(4 genes down-regulated)**			
***Pentose phosphate pathway***				***Carbohydrate metabolism***			
At2g21330	fructose-bisphosphate aldolase 1	1.3	0.4	At1g70730	phosphoglucomutase, cytoplasmic	-0.7	-0.32
***Fatty acid beta oxidation***				***Stress response***			
At5g09660	peroxisomal malate dehydrogenase 2	1.4	1.0	At3g11930	universal stress protein (USP)	-2.6	-2.8
***Interconversion of CO2 and bicarbonate***				At5g24770	vegetative storage protein 2 (VSP2)	-2.4	-3.4
At3g01500	carbonic anhydrase 1, chloroplast	3.3	1.8	At5g24780	vegetative storage protein 1 (VSP1)	-2.9	-3.5
At5g14740	carbonic anhydrase 2, cytoplasm	1.4	1.2				
***Protein folding***				***P. novae-zelandiae *(8 genes up-regulated)**			
At1g55490	chloroplast chaperonin 60 beta	0.9	1.4	***Detoxification of xenobiotics***			
At5g20720	20 kDa chaperonin chloroplast	0.9	2.1	At1g17170	glutathione S-transferase	2.7	9.7
***Oxidative stress***				At3g62700	glutathione-conjugate transporter	1.2	3.2
At1g07890	L-ascorbate peroxidase 1, cytosolic	0.8	0.4	***Cellular metal ion homeostasis***			
At5g06290	2-cys peroxiredoxin, chloroplast	1.1	1.2	At4g16370	oligopeptide transporter family protein	1.4	8.5
***Serine family amino acid metabolic process***				***Interconversion of CO2 and bicarbonate***			
At4g11640	serine racemase	2.3	1.6	At1g23730	carbonic anhydrase	1.7	4.3
***Other***				***Stress response***			
At4g02530	chloroplast thylakoid lumen protein	1.3	3.5	At5g24770	vegetative storage protein 2 (VSP2)	3.5	12.1
At1g54780	thylakoid lumen 18.3 kDa protein	1.3	1.3	At5g24780	vegetative storage protein 1 (VSP1)	2.6	6.3
At2g37220	chloroplast RNA binding protein	1.0	3.7	At5g58390	peroxidase	1.4	3.5
At1g57770	amine oxidase family	1.1	2.2	***Other***			
At1g62750	translation elongation factor	1.2	1.6	At1g79690	nudix hydrolase homolog 3	0.9	2.9
At3g15360	thioredoxin M-type 4	1.5	4.6				
At5g19440	alcohol dehydrogenase	0.9	2.2				

Locus, description, log fold change (logFC), and gene ontology terms (biological process) for genes found specifically up- and down-regulated in *P. cheesemanii*, *P. exile *and *P. novae-zelandiae *when compared to the two remaining species combined. Only genes for which both transcripts (T) and proteins (P) have been found to be differentially expressed are listed (*P. cheesemanii*: 29 up-regulated genes, 2 down-regulated genes; *P. exile*: 4 up-regulated genes, 4 down-regulated genes; *P. novae-zelandiae*: 8 up-regulated genes; compare Venn diagrams in left panel of figure 1).

### Glucosinolate profiles and hydrolysis products

To test how faithfully both transcript and protein expression patterns would predict physiological differences between species, we examined a trait, which we had previously shown to vary greatly in *Pachycladon*, namely, glucosinolate biosynthesis and hydrolysis [3]. Glucosinolate biosynthesis and hydrolysis genes found to be differentially regulated in the twelve comparisons by either transcript or protein profiling are summarized in table 4.

**Table 4:** Glucosinolate metabolism loci up-regulated in transcript and protein profiling

Differentially expressed transcripts				Differentially expressed proteins			
**CH up**	**CH down**	**CH up**	**CH up**	**CH up**	**CH down**	**CH up**	**CH up**
**EX+NZ down**	**EX+NZ up**	**EX down**	**NZ down**	**EX+NZ down**	**EX+NZ up**	**EX down**	**NZ down**

At4g03050	*At1g54000*	At4g03050	At4g03050		At5g14200	**At2g43100**	
At4g03060		**At4g03060**	At4g03060				
			At5g23010				
			At2g43100				

**EX up**	**EX down**	**EX up**	**EX up**	**EX up**	**EX down**	**EX up**	**EX up**
**CH+NZ down**	**CH+NZ up**	**CH down**	**NZ down**	**CH+NZ down**	**CH+NZ up**	**CH down**	**NZ down**

*At3g14210*	At4g03060	*At1g54000*	At5g23010	*At1g54010*	**At2g43100**		
			*At3g14210*		*At3g14220*		
			*At1g54030*		*At1g54040*		

**NZ up**	**NZ down**	**NZ up**	**NZ up**	**NZ up**	**NZ down**	**NZ up**	**NZ up**
**CH+EX down**	**CH+EX up**	**CH down**	**EX down**	**CH+EX down**	**CH+EX up**	**CH down**	**EX down**

*At1g54000*	At5g23010	*At1g54000*		**At2g43100**		**At2g43100**	**At2g43100**
	At2g43100			**At5g14200**		**At5g14200**	**At5g14200**
	At4g03050			**At1g31180**		**At1g31180**	*At1g54040*
	*At1g54030*			*At1g54040*		***At1g54040***	*At1g54030*
	*At3g14210*			*At1g54030*		*At1g54030*	*At3g14220*
				*At3g14220*		*At3g14220*	

**Figure 2:**
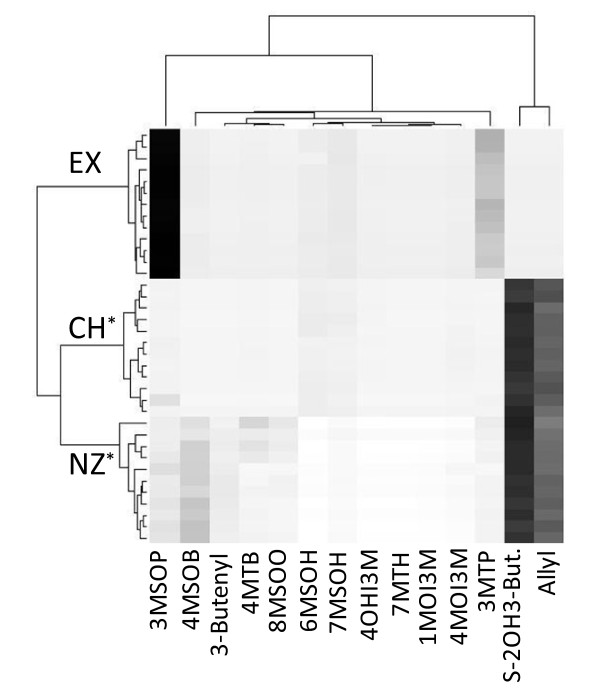
**Hierarchical clustering of glucosinolate profiles**. Fourteen compounds were identified across individuals of *P. cheesemanii *(CH, n = 12), *P. exile *(EX, n = 13) and *P. novae-zelandiae *(NZ, n = 12). Data used for hierarchical clustering represent proportions from total contents; for comparisons of concentrations see additional file 2, figure S1. Note that CH and NZ share their two major compounds allyl and S-2OH3-butenyl glucosinolate despite being less closely related than CH and EX. *The glucosinolate profiles for *P. novae-zelandiae *and *P. cheesemanii *were independently confirmed during the analysis of glucosinolate breakdown products in both species (table 5). Abbreviations: 3MTP, 3-methylthiopropyl glucosinolate; 4MTB, 4-methylthiobutyl glucosinolate; 3MSOP, 3-methylsulfinylpropyl glucosinolate; 4MSOB, 4-methylsulfinylbutyl glucosinolate; allyl, 2-propenyl glucosinolate; 3-butenyl, 3-butenyl glucosinolate; S-2OH3-butenyl, *S*-2-hydroxy-3-butenyl glucosinolate; 6MSOH, 6-methylsulfinylhexyl glucosinolate; 7MTH, 7-methylthioheptyl glucosinolate 7MSOH, 7-methylsulfinylheptyl glucosinolate; 8MSOO, 8-methylsulfinyloctyl glucosinolate; 1MOI3M, 1-methoxy-indolyl-3-methyl glucosinolate; 4OHI3M, 4-hydroxy-indolyl-3-methyl glucosinolate, 4MOI3M, 4-methoxy-indolyl-3-methyl glucosinolate.

Loci involved in glucosinolate biosynthesis and *hydrolysis *(italicised) found up-regulated in each of the twelve comparisons by either transcript or protein profiling. Glucosinolate biosynthesis: At4g03050 (oxoglutarate dependent dioxygenase 3, AOP3) and At4g03060 (oxoglutarate dependent dioxygenase 2, AOP2) catalyze the accumulation of hydroxyalkyl and alkenyl glucosinolates, respectively. At5g23010 (methylthioalkylmalate synthase), At2g43100 (methylthioalkylmalate isomerase), At5g14200 (methylthioalkylmalate dehydrogenase), and At1g31180 (methylthioalkylmalate dehydrogenase) are involved in the chain elongation of methionine and thus can be considered indicators for the accumulation of C4 glucosinolates as opposed to C3 glucosinolates. *Glucosinolate hydrolysis: *At3g14210 (epithiospecifier modifier 1, ESM1) and At1g54040 (epithiospecifier proteins, ESP) determine the outcome of glucosinolate hydrolysis in that ESP is necessary for nitrile formation whereas ESM1 promotes the formation of isothiocyanates. Other myrosinase-binding/associated proteins (At1g54000, At1g54010, At1g54030, At3g14220) whose biological function remains to be determined are also found up-regulated in some of the species. Loci whose expression is in agreement with profiles of glucosinolates (figure 2, additional file 2, figure S1) and glucosinolate hydrolysis products (table 5) are highlighted in bold.

Expectations for glucosinolate profiles were that *P. cheesemanii *would have higher levels of alkenyl and hydroxyalkyl glucosinolates (suggested by up-regulation of AOP2 and AOP3 transcript levels) than *P. exile *and *P. novae-zelandiae *and *P. cheesemanii *would have higher levels of four carbon (C4) glucosinolates than *P. exile *(suggested by the up-regulation of methylthioalkylmalate isomerase protein levels, MAM-I, At2g43100) (table 4). Conflicting predictions were made for *P. novae-zelandiae *since the up-regulation of methionine chain elongation proteins, such as MAM-I (At2g43100) and two proteins of methylthioalkylmalate dehydrogenase (MAM-D, At5g14200, At1g31180, [14]) suggests higher levels of C4 glucosinolates in *P. novae-zelandiae *than in the other two species. However, up-regulation of MAM-I and methylthioalkylmalate synthase (MAM1, At5g23010) transcript levels in *P. cheesemanii *when compared to *P. novae-zelandiae *suggests higher levels of C4 glucosinolates in the former (table 4). Contradictory predictions were also derived for *P. exile*, as up-regulation of MAM1 transcripts indicated higher C4 levels in *P. exile *than in *P. novae-zelandiae *and down-regulation of MAM-I proteins suggested higher levels of C4 glucosinolates in *P. novae-zelandiae *and *P. cheesemanii *when compared to * P. exile *(table 4). To verify these predictions we analyzed glucosinolate profiles in the same specimens that had been used for transcript and protein profiling. Fourteen compounds were identified across the three species (additional file 2, figure S1). Interestingly, *P. cheesemanii *and *P. exile *had very distinct glucosinolate profiles whereas *P. cheesemanii *and *P. novae-zelandiae*, despite being less closely related than *P. cheesemanii *and *P. exile*, shared their two major compounds allyl and *S *-2-hydroxy-3-butenyl glucosinolate (figure 2). This high similarity in glucosinolate profiles between *P. cheesemanii *and *P. novae-zelandiae *was also surprising given that *P. cheesemanii *and *P. exile *had been found to have more similar transcript and protein profiles than *P. cheesemanii *and *P. novae-zelandiae *(table 1). *P. exile *did produce almost no alkenyl glucosinolates (allyl, 3-butenyl, *S*-2-hydroxy-3-butenyl combined) and *P. cheesemanii *and *P. novae-zelandiae *did not differ significantly in their production of alkenyl glucosinolates (85 μmol/g vs. 77.8 μmol/g, p value >0.05) (additional file 2, figure S1). Thus the prediction from AOP2 gene expression suggesting higher levels of alkenyl glucosinolates in *P. cheesemanii *when compared to *P. exile *and *P. novae-zelandiae *was only fulfilled for *P. exile*. Hydroxy alkenyl glucosinolates were not detected in any of the three species contrary to the up-regulation of AOP3 transcripts in *P. cheesemanii*. As previously discussed [3], AOP3 expression might be an artefact of cross-hybridization of AOP2 transcripts with the AOP3 probe and as such constitute a drawback of using a heterologous microarray. *P. novae-zelandiae *however produced slightly more C4 glucosinolates than *P. cheesenmanii *(59 μmol/g vs 48 μmol/g, p value = 0.059, additional file 2, figure S1) and also accumulated three additional C4 glucosinolates (4-methylthiobutyl, 4-methylsulfinylbutyl, 3butenyl) compared to *P. cheesemanii *which only accumulated the C4 compound *S*-2-hydroxy-3 butenyl glucosinolate (figure 2). *P. exile *did not produce any C4 glucosinolates. Thus the differential expression of MAM-I and the two MAM-D proteins (up in *P. novae-zelandiae*, up in *P. cheesemanii*, down in *P. exile*) proved to be a reliable indicator of metabolite profiles whereas up-regulation of MAM-I transcripts in *P. cheesemanii *and MAM1 transcripts in *P. exile *did not. Neither array nor protein profiling captured the expression of 2-oxoacid-dependent dioxygenase which has recently been shown to catalyze the production of 2-hydroxy-3-butenyl glucosinolates from 3-butenyl glucosinolates [15] and which we predict to be up-regulated in *P. cheesemanii *and *P. novae-zelandiae *when compared with *P. exile*.

Expectations for glucosinolate hydrolysis were that during glucosinolate hydrolysis *P. exile *would produce isothiocyanates (predicted by up-regulation of ESM1 transcripts, At3g14210, and down-regulation of ESP proteins, At1g54040) and *P. novae-zelandiae *would produce nitriles (predicted by strong up-regulation of the ESP protein, At1g54040 and the down-regulation of ESM1 transcripts, At3g14210) (table 4). There were no particular expectations for *P. cheesemanii *although in the absence of myrosinase-associated enzymes, glucosinolates will most likely convert into isothiocyanates. Interestingly, transcript and protein profiling revealed other myrosinase-associated genes to be up-regulated in *P. exile *(At1g54000, At1g54010, At1g54030) and *P. novae-zelandiae *(At1g54000, At1g54030, At3g14220) and down-regulated in *P. cheesemanii *(At1g54000), *P. exile *(At3g14220) and *P. novae-zelandiae *(At1g54030) (table 4). All of these additional loci are located in close proximity to ESP (At1g54040) or ESM1 (At3g14210) in *A. thaliana*. Due to being recently classified as nationally critical in New Zealand, no plants of *P. exile *were available for glucosinolate hydrolysis analysis. However, the comparison of glucosinolate hydrolysis products of *P. cheesemanii *and *P. novae-zelandiae*, confirmed our predictions. Both species have the same major glucosinolates but whereas in *P. cheesemanii *all compounds hydrolyzed into isothiocyanates, in *P. novae-zelandiae*, the two major compounds were converted into their corresponding nitriles and epithionitriles (table 5). This ESP-associated polymorphism in glucosinolate hydrolysis products had been found earlier in two other closely related *Pachycladon *species, namely *P. enysii *(up-regulates ESP and produces nitriles) and *P. fastigiatum *(up-regulates ESM1 and produces isothiocyanates) [3]. However, *P. enysii *and *P. fastigiatum's *glucosinolate profiles were different from those of *P. cheesemanii *and *P. novae-zelandiae *in that both of the latter produced S-2-hydroxy-3-butenyl, a compound neither present in *P. enysii *nor *P. fastigiatum*. The strong differential expression of myrosinase-associated transcripts and proteins is consistent with the hypothesis that glucosinolate hydrolysis has been under selection during the divergence of *Pachycladon *species.

**Table 5:** Glucosinolate hydrolysis products (nmol/g fresh weight, mean ± SD) of *P. novae-zelandiae *and *P. cheesemanii*

Glucosinolates	Isothiocyanates			Nitriles/epithionitriles		
	compound	mean ± sd	%	compound	mean ± sd	%
***P. novae-zelandiae***						
*S*-2-OH-3-butenyl	goitrin	1394 ± 698	12.4	2OH3but-CN	590 ± 100	5.3
				epithio-2OH3B-I	2446 ± 397	21.8
				epithio-2OH3B-II	2269 ± 371	20.2
allyl	allyl-ITC	1622 ± 668	14.4	epithio-allyl	1868 ± 450	16.6
3-butenyl	3-but-ITC	162 ± 45	1.4	-	-	
3MTP	3MTP-ITC	449 ± 226	4	3MTP-CN	67 ± 27	0.6
3MSOP	3MSOP-ITC	30 ± 41	0.3	-	-	
4MTB	4MTB-ITC	210 ± 122	1.9	4MTB-CN	22 ± 14	0.2
4MSOB	4MSOB-ITC	58 ± 48	0.5	-	-	
8MTO	8MTO-ITC	27 ± 9	0.2	-	-	
9MTN	9MTN-ITC	28 ± 6	0.2	-	-	
	**all**		**35.3**	**all**		**64.7**

***P. cheesemanii***						
*S*-2-OH-3-butenyl	goitrin	5115 ± 981	60.7	2OH3but-CN	32 ± 15	0.4
allyl	allyl-ITC	3261 ± 997	38.7	epithio-allyl	4 ± 2	0.1
3-butenyl	3-but-ITC	9 ± 4	0.1	-	-	
	**all**		**99.5**	**all**		**0.5**

Note that *P. novae-zelandiae *is capable of converting its main glucosinoalates (*S*-2-hydroxy-3-butenyl and allyl) into its corresponding nitriles ((S)-3-hydroxy-pent-4-enenitril) and epithionitriles (diastereomers 1 and 2 of (S)-3-hydroxy-3-((R, S)-thiiran-2-yl)propanenitril, 2-((*R, S*)-thiiran-2-yl)ethanenitril) whereas *P. cheesemanii *is not. Despite minor glucosinolates being converted to isothiocyanates in *P. novae-zelandiae*, 65% of its glucosinolate breakdown products are nitriles/epithionitriles. In *P. cheesemanii*, 99.5% of glucosinolates are converted to isothiocyanates indicating nitrile/epithionitrile forming activity in *P. novae-zelandiae *but not *P. cheesemanii*.

Abbreviations: goitrin, goitrin; allyl-ITC, 2-propenylisothiocyanate; 3-but-ITC, 3-butenylisothiocyanate; 3MTP-ITC, 3-methylthiopropylisothiocyanate; 3MSOP-ITC, 3-methylsulfinylpropylisothiocyanate; 4MTB-ITC, 4-methylthiopropylisothiocynate; 4MSOB-ITC, 4-methylsulfinylbutylisothiocyanate; 8MTO-ITC, 8-methylthiooctylisothiocyanate; 9MTN-ITC, 9-methylthiononyl-isothiocyanate; 2OH3but-CN, *S*-3-hydroxy-pent-4-enenitrile; epithio-2OH3B-I, diastereomer 1 of (*S*)-3-hydroxy-3-((*R, S*)-thiiran-2-yl)propanenitril; epithio-2OH3B-II, diastereomer 2 of (*S*)-3-hydroxy-3-((*R, S*)-thiiran-2-yl)propanenitril; epithio-allyl, 2-((*R, S*)-thiiran-2-yl)ethanenitril; 3MTP-CN, 4-methylthiobutanenitrile; 4MTB-CN, 5-methylthiopentanenitrile.

In summary, predictions of glucosinolate metabolism from protein profiling were more accurate than predictions from transcript profiling. Based on protein profiles we predicted that *P. novae-zelandiae *would accumulate more C4 glucosinolates than the other two species and would produce nitriles in hydrolysis instead of isothiocyanates. Both predictions were confirmed. In contrast, the prediction from transcript profiles that *P. cheesemanii *would produce more alkenyl glucosinolates than *P. novae-zelandiae *and *P. exile *was only validated for *P. exile*.

## Discussion

In this study we compared protein profiles with transcript profiles for three species of New Zealand alpine *Pachycladon*. We employed a heterologous approach to obtain both transcript and protein profiles using *Arabidopsis *microarrays for transcript quantification and the *Arabidopsis *genome as a reference for the quantification of peptide spectra. Such a heterologous approach may limit the number of transcripts and proteins that can be unambiguously identified and whose expression can be reliably quantified. Thus we cannot rule out that these limitations contributed to some of the incongruence we found between transcript and protein patterns. However, the fact that we obtained both transcript and protein data for a set of 1074 genes and that we found significant correlations and overlap in differential transcript and protein expression demonstrates that the benefits of using heterologous approaches outweigh their drawbacks. Heterologous microarray studies have long been perceived as a valuable tool for the study of species diversity [16]. Our study is one of the first that combines the use of heterologous microarrays with heterologous shotgun proteomics in the context of ecological prediction.

We adopted two approaches to estimate congruence between transcript and protein profiles. First, we determined 'overall congruence' through correlations of transcript and protein abundance for a set of 1074 genes surveyed by both microarray analyses and shotgun proteomics. These correlations differed between species and the highest correlation was found in *P. cheesemanii *(0.52). The coefficients we observed were consistent with those found in other, non-plant systems (0.2-0.5, [17,18]). We then determined congruence in differential expression by investigating down- stream prediction (from transcriptome to proteome) and up-stream prediction (from proteome to transcriptome). We found that transcript patterns predicted protein patterns best in *P. novae-zelandiae *(30.2%), followed by *P. exile *(20%) and *P. cheesemanii *(19.2%). Up-stream prediction was on average much weaker (12.6%) than down-stream prediction (23.6%), and the results differed markedly between the three species (*P. cheesemanii *24.5%, followed by *P. exile *10% and *P. novae-zelandiae *3.2%). Given considerable incongruence of transcript and protein patterns, we suggest that for the prediction of physiologies consideration needs to be given to both transcript and protein profiles. Although more minor in scope than transcript profiling, protein profiling not only proved to be a valuable tool for validation of transcript profiling but, in this study, was also a more accurate predictor of metabolite patterns than transcript profiling.

Interestingly, proteomes were more dissimilar between species than transcriptomes. This may be due to different protein turnover rates in these species, or it may be explained as a consequence of a greater divergence of the species' proteomes. In other words, depending on down-stream processes such as translation, post-transcriptional modification and protein degradation, relatively similar transcription profiles may give rise to relatively dissimilar protein profiles. Thus small differences in transcript levels may manifest themselves in large differences in protein levels and vice versa, which may also be an explanation for the relatively large degree of discordance between transcript and protein profiles. Transcript and protein profiles largely mirrored inferred phylogenetic relatedness among species [8,9] with the profiles of *P. cheesemanii *and *P. exile *being more similar to each other than each was to the profiles of *P. novae-zelandiae*. Interestingly, *P. cheesemanii *and *P. novae-zelandiae *had more similar glucosinolate profiles than did *P. cheesemanii *and *P. exile*. The former species shared their main two compounds allyl and *S*-2-hydroxy-3-butenyl. *P. exile *neither produced alkenyl nor C4 glucosinolates, thus its profile is most similar to that of the *P. fastigiatum *chemotype 2 [3]. In contrast to *P. cheesemanii *and *P. exile*, *P. novae-zelandiae *produced a complex blend of glucosinolates. The finding that similarity patterns in glucosinolate profiles do not correspond to phylogenetic relationships might be explained by selection for glucosinolate metabolism during diversification of *Pachycladon *species, and is an hypothesis that requires further investigation.

In the present study, a number of candidate genes have been identified as having been potentially important during diversification of *Pachycladon *species. These include myrosinase-binding proteins two of which, namely, ESP (At1g54040) and ESM1 (At3g14210) have been shown to direct glucosinolate hydrolysis towards nitriles and isothiocyanates and thus directly affect the toxicity and palatability of plant tissue to herbivores [19,20]. We found the ESP protein up-regulated in *P. novae-zelandiae *(and confirmed nitrile- and epithionitrile-specifying activity) along with genes that in *A. thaliana *are physically linked to ESP, such as At1g54030 and At1g54000, as well as At3g14220 which is physically linked to ESM1. In *P. exile*, there was evidence for up-regulation of At1g54000, At1g54010, At1g54030 and ESM1. Given previous observations [3] of up-regulation of ESM1 in *P. fastigiatum *(and confirmed isothiocyanate-specifying activity), At1g54030 and At1g54020 in *P. fastigiatum *and ESP in *P. enysii *(and confirmed nitrile-specifying activity), we hypothesize that at least two gene clusters have been under selection during Pleistocene species radiation of *Pachycladon *[8]. These two clusters correspond to five loci on *A. thaliana *chromosome one (At1g54000-At1g54040) and two loci on chromosome three (At3g14210, At3g14220) which are all annotated as myrosinase-associated proteins. We have begun to characterize both clusters in several *Pachycladon *species regarding their gene structure and nucleotide diversity patterns.

Other genes of potential adaptive significance in *Pachycladon *are genes which mediate differences in the glucosinolate profiles of *Arabidopsis*. These include methylthioalkylmalate synthase (At5g23010), methylthioalkylmalate isomerases (At2g43100, At3g58990) and methylthioalkylmalate dehydrogenases (At5g14200, At1g31180), which specify side chain lengths of aliphatic glucosinolates and oxo-acid-dependent-dioxygenases which catalyze the synthesis of alkenyl (At4g03060) and hydroxyalkenyl (At2g25450) glucosinolates. Their potential importance in divergence of *Pachycladon *species is suggested in the present study by the up-regulation of At2g43100, At1g31180 and At5g14200 in *P. novae-zelandiae *and of At4g03060 and At2g43100 in *P. cheesemanii*. Previous findings of up-regulation of At5g23010, At2g43100, At3g58990, At5g14200, At1g31180, At4g03060 in *P. enysii *[3], as well as the presence of strikingly different glucosinolate profiles among *Pachycladon *species (this study and [3]) are also consistent with an hypothesis of adaptive diversification of glucosinolate metabolism in *Pachycladon*.

In addition to the potential significance of glucosinolate biosynthesis and hydrolysis genes during *Pachycladon *diversification, we observed expression patterns that may be indicative of ecophysiological differences among *Pachycladon *species. For example, the up-regulation of different beta carbonic anhydrase genes in *P. cheesemanii *(At3g01500, AtβCA1, chloroplast-located, At5g14740, AtβCA2, cytosol-located, [13]) and *P. novae-zelandiae *(At1g23730, AtβCA3, cytosol-located, [13]) suggests differential recruitment of carbonic anhydrases during *Pachycladon *diversification and regulation of physiological processes requiring the interconversion of carbon dioxide and bicarbonate. Chloroplast isoforms may contribute to maintenance of adequate carbon dioxide concentration for Rubisco whereas cytoplasmic isoforms may provide bicarbonate to phosphoenolpyruvate decarboxylase [21]. This is the primary carboxylating enzyme in C4 and CAM plants and is also an enzyme replenishing tricarboxylic acid cycle intermediates in leaves of C3 plants [21]. Reports of co-localization of carbonic anhydrases with phosphoenolpyruvate decarboxylase and pyruvate kinase in roots indicate a role of carbonic anhydrases in re-assimilation of carbon dioxide into metabolic intermediates also in non-photosynthetic tissues [21]. Thus the differential expression of three beta carbonic anhydrases suggests that *P. novae-zelandiae *and *P. cheesemanii *may regulate carbon dioxide and bicarbonate supply to Rubisco and phosphoenolpyruvate decarboxylase differently.

Interestingly, a suite of genes up-regulated in *P. cheesemanii *have recently been found important in the drought responses of *Populus *genotypes that exhibit contrasting leaf carbon isotope discrimination measurements (an estimate of intrinsic water use efficiency, [22]). These are genes involved in oxidative stress (ascorbate peroxidase and 2 cys peroxiredoxin) and protein folding (20kDa chloroplastic chaperonin (table 3, [22]). Other genes involved in drought response in *Populus *were genes involved in carbon fixation (chloroplast glyceraldehyde-3-phosphate dehydrogenase B), photosynthesis (oxygen evolving enhancer 1), ATP synthesis (chloroplast ATP synthase alpha) and protein folding (heat shock cognate 70kDa protein). Up-regulated in *P. cheesemanii *was a similar set of genes: for carbon fixation (chloroplast glyceraldehyde-3-phosphate dehydrogenase A subunit 2), photosynthesis (oxygen evolving enhancer 3), ATP synthesis (chloroplast ATP synthase gamma) and protein folding (chaperonin 60). In *Populus*, abundances of some proteins, amongst them a mitochondrial succinyl CoA ligase protein, was found to be correlated with leaf carbon isotope discrimination [22]. The same gene is among the few genes specifically up-regulated in *P. exile *(table 3). Oxidative stress genes and chloroplast-localized molecular chaperones have also been found up-regulated or constitutively higher expressed in drought resistant genotypes of potato [23] and barley [24]. Among these were homologues of glutathione-S-transferase and glutathione transporters which were also up-regulated in *P. novae-zealandiae *as well as a homologue of thioredoxin which was up-regulated in *P. cheesemanii*. Given that many elements of the transcriptomic and proteomic signatures of drought response in *Populus, Solanum *and *Hordeum *are found in differential transcript and protein patterns of *Pachycladon*, we hypothesize differences in drought resistance and water use efficiency between *Pachcyladon *species. These differences may have evolved in response to differences in soil water availability between species' habitats. Based on our findings we have initiated comparisons of carbon dioxide assimilation rates and carbon isotope discrimination to characterize photosynthesis and water use efficiency in *Pachycladon*. Furthermore, efforts are underway to measure soil moisture levels and other environmental parameters at native *Pachycladon *sites.

Other genes potentially interesting for species diversification in *Pachycladon *are serine racemase (At4g11640, up-regulated in *P. cheesemanii *) and two vegetative storage proteins (At5g24770, At5g24780, up-regulated in *P. novae-zelandiae*, down -regulated in *P. exile *). However, their biological roles in plant metabolism are not fully understood, hindering the interpretation of their differential expression in *Pachyladon *in the context of diversification. Serine racemase is primarily expressed in elongating and developing cells such as root tips, developing leaves and shoot meristems in *A. thaliana *[25]. The enzyme has been demonstrated to have a higher dehydration than racemisation activity leading to the hypothesis that it has a role in providing pyruvate from serine in highly dividing cells. Pyruvate is a key metabolic intermediate of many pathways, such as glycolysis and the biosynthesis of amino acids, organic acids and fatty acids. Thus, similarly to carbonic anydrase, serine racemase may act in the regeneration of metabolic intermediates. One of the vegetative storage proteins (At5g24770) has been shown to have an 'anti-insect' activity that is associated with its phosphatase activity, and is primarily expressed in *Arabidopsis *flowers. It is induced by wounding, methyl jasmonate, phosphate deprivation and insect feeding and assumed to also play a role in temporary amino acid storage [26]. Given these multiple roles in abiotic and biotic stress responses, the preferential expression in *P. novae-zelandiae *may confer this species an advantage in coping with stress compared to *P. exile *and *P. cheesemanii*.

## Conclusion

In summary, although shotgun proteomics provided information on fewer genes than transcript profiling, it provided a valuable means for validating high throughput gene expression data and predicting physiological attributes of plants. The combined application of transcript and protein profiling has allowed us to identify candidate genes potentially important in adaptive diversification. In *Pachycladon *we identified genes with confirmed or predicted roles in glucosinolate biosynthesis and hydrolysis, carbon dioxide bicarbonate conversion, water use efficiency and general stress-related processes. From these gene sets we predict that adaptation to herbivores and pathogens and soil moisture have been important in driving diversification of *Pachycladon *species. Differences in photosynthesis and carbon fixation may indicate selection for different growth rates. Characterization of gene clusters, physiological and habitat differences in *Pachycladon *are underway to follow up predictions from this comparative profiling study. Microarray analyses of *Pachycladon *roots are also underway to help test the hypothesis that adaptation to different soil types has been a major driver of diversification in *Pachycladon*.

## Methods

### Sampling

Five *Pachycladon *species were initially sampled (*P. cheesemanii*: accession Bob's cove, CH; *P. exile*: accession Awahokomo, EX; *P. novae-zelandiae*: accession Old man range, NZ; *P. fastigiatum*, accession Serpentine creek, FA; *P. enysii*, accession: Avalanche peak, PE). Seeds were stratified at 4°C for a week and then germinated on potting soil in early July 2007. Plants were grown in shade houses at Landcare Research (Lincoln, NZ) for seven months. In February 2008, roots and above-ground plant tissues of each plant were separately flash frozen and ground in liquid nitrogen. Ground material was partially allocated to RNA analysis and partially freeze-dried for protein and metabolite analysis. Replicate plant numbers were as follows: CH, n = 12; EX, n = 13; NZ, n = 12; FA, n = 8; EN, n = 8. *P. cheesemanii*, *P. exile *and *P. novae-zelandiae *were grown until all three species started producing their first seeds. By sampling leaves, flowers and siliques we expected to increase the probability of capturing expression differences. *P. fastigiatum *and *P. enysii *were harvested in rosette stage to enable a valid comparison with a previous field study [3]. Here we will only report the results for the comparisons of the three fruiting species (CH, EX, NZ) whereas the results for the *P. enysii *- *P. fastigiatum *comparison will be reported elsewhere.

Five descendants of one *P. cheesemanii *(from Bob's cove) and one *P. novae-zelandiae *(from Old man range) individual were grown in growth chambers (MPI for Chemical Ecology, Jena, Germany) under *Arabidopsis *growing conditions as previously described [3] for the analysis of glucosinolate hydrolysis products. *P. exile *was not available for the follow up experiment on glucosinolate hydrolysis products since its recently changed conservation status to 'nationally critical' [12] prevented the collection of further plants in the wild.

### Microarray analysis

RNA was isolated, quantified and quality checked as described in [3] separately for each plant and subsequently pooled for each species. CDNA synthesis, labelling and hybridization were done according to [27]. An *Arabidopsis thaliana *genome-wide array (spotted with the AROS version 1.0 genome set available from Operon Biotechnologies) was provided by Plant and Food Research (Auckland, NZ). The array had been successfully used in heterologous hybridizations involving FA and EN previously [3]. Hybridizations with DNA from *P. enysii*, *P. fastigiatum *and *P. novae-zelandiae *yielded no differences in signal intensities across species [3]. Moreover, sequence divergence between *P. exile *and *P. fastigiatum*, the most distant species in the *Pachycladon *radiation, has been found to be less than one per cent (0.0088 ± 0.0016 substitutions/site, unpublished data). The diversification of *Pachycladon *has been estimated to have occurred within the last 0.8 mya [8] which is consistent with the low level of genetic divergence between species. For these reasons, we did not expect the genetic distance between individual *Pachycladon *genomes to contribute to the differences in gene expression measured using *A. thaliana *microarrays. A total of 36 two channel microarrays were hybridized: five root samples were hybridized in a loop-wise fashion using ten arrays; five leave samples were hybridized in a loop-like fashion using fourteen arrays; five root and three leaf samples were hybridized in a loop-like fashion using twelve arrays (additional file 3, table S2). Each sample was thus hybridized multiple times while being labelled either with Cy3 or Cy5 (additional file 3, table S2). Data analysis used the R-based package limma [28]. The statistical analysis made use of three loop designs (additional file 3, table S2) involving additional replicates for some species. All 36 arrays were subjected to data pre-processing, e.g. filtering of low intensity signals, background correction, within array normalization and between array normalization. Linear models were fitted to the log2 Cy5/Cy3 ratios separately for each gene. Since the focus of this study was on CH, EX and NZ, the number of differentially expressed genes was calculated for six contrasts. These contrasts involve all pair wise comparisons between the three species (CH vs. EX, EX vs. NZ, CH vs. NZ) as well as comparisons of each species against the remaining two species combined (CH vs. EX+NZ, EX vs. CH+NZ, NZ vs. CH+EX). Each of the six contrasts was determined three times using different pre-processing parameters (data not shown) and the number of differentially expressed genes was determined conservatively by reporting only those genes that had been identified as differentially expressed in all three analyses. P-values were adjusted using the method by Benjamini and Hochberg [29] as implemented in limma. Microarray data have been deposited at the Gene Expression Omnibus genomics data repository hosted by NCBI (GEO accession nr: GSE19017).

### Shotgun proteomics

Three randomly selected plants of each species (CH, EX, NZ, FA, EN) were selected to provide triplicates for proteome analysis.

#### Protein extraction and SDS-PAGE

20 mg freeze dried leaf powder was suspended in 600 μl of 10% TCA in acetone, 0.07% β-mercaptoethanol, and incubated at -20°C for 45 minutes. The extract was centrifuged for 30 minutes at 16,000 × g, and the pellet was collected and washed with 600 μl 100% acetone followed by the centrifugation for 30 minutes at 16,000 × g. The acetone washing step was repeated three times for the complete removal of pigments, lipids and other lipophilic molecules. The colourless resulting pellet was lyophilized in a vacuum centrifuge and protein quantification was performed by Bradford assay. Extracted proteins in sodium dodecyl sulfate (SDS) sample buffer (50 μg per well) were separated on 7.5% Bis-Tris polyacrylamide gels at 180 V for 1 h. After electrophoresis, proteins were visualized using Coomassie Blue. The gels were then washed twice in water (10 min per wash), before individual lanes were cut into 16 slices of equal sizes from top to bottom. Proteins in each slice were reduced, alkylated and subjected to trypsin digestion as previously described [30].

#### Nanoflow liquid chromatography - tandem mass spectrometry

The tryptic digest extracts from 1DE gel slices were analyzed by nanoLC-MS/MS using a LTQ-XL ion-trap mass spectrometer (Thermo, CA, USA). Reversed phase columns were packed in-house to approximately 7 cm (100 μm i.d.) using 100 Å, 5 mM Zorbax C18 resin (Agilent Technologies, CA, USA) in a fused silica capillary with an integrated electrospray tip. A 1.8 kV electrospray voltage was applied via a liquid junction up-stream of the C18 column. Samples were injected onto the C18 column using a Surveyor autosampler (Thermo, CA, USA). Each sample was loaded onto the C18 column followed by initial wash step with buffer A (5% (v/v) ACN, 0.1% (v/v) formic acid) for 10 min at 1 μL min^-1^. Peptides were subsequently eluted from the C18 column with 0%-50% Buffer B (95% (v/v) ACN, 0.1% (v/v) formic acid) over 58 min at 500 nL min^-1 ^followed by 50-95% Buffer B over 5 min at 500 nL min^-1^. The column eluate was directed into a nanospray ionization source of the mass spectrometer. Spectra were scanned over the range 400-1500 amu. Automated peak recognition, dynamic exclusion, and tandem MS of the top six most intense precursor ions at 35% normalization collision energy were performed using the Xcalibur software (version 2.06) (Thermo, CA, USA) [30].

#### Protein identification

Raw files were converted to mzXML format and processed through the global proteome machine (GPM) software using version 2.1.1 of the X!Tandem algorithm, freely available from http://www.thegpm.org[31,32]. For each experiment, the 16 fractions were processed sequentially with output files for each individual fraction, and a merged, non-redundant output file was generated for protein identifications with log (e) values less than -1. MSMS spectra were searched against the *A. thaliana *protein database within GPM containing 30480 protein sequences as of April 2008. The database also incorporated common human and trypsin peptide contaminants. The search was performed against a reversed sequence database to evaluate the false discovery rate (FDR). Search parameters included MS and MS/MS tolerances of ± 2 Da and ± 0.2 Da, tolerance of up to 3 missed tryptic cleavages and K/R-P cleavages. Fixed modifications were set for carbamidomethylation of cysteine and variable modifications were set for oxidation of methionine. Only proteins that were present in all replicates and had a minimum of 2 peptides were included in further analysis. After filtering, protein level false discovery rates were calculated at less than 1% (M. Mirzaei, D. Pascovici, T. Keighley, C. Voelckel, P. B. Heenan, P. A. Haynes: Shotgun proteomic profiling of five species of New Zealand *Pachycladon*, submitted).

#### Quantitative proteomic analysis

Protein abundance data were calculated based on normal spectral abundance factor (NSAF) values as described previously [7]. For each sample, *i*, the number of spectral counts (*SpC *) identifying a protein, *k*, was divided by the molecular weight of the protein in kDa. (SpC/MW)_*k *_values were then divided by the sum of (SpC/MW) for all (*N *) proteins in the experiments to give the NSAF_*i *_values. Spectral counts identifying more than one isoform of a gene locus were combined and expressed as NSAF per locus. For each protein *k*, the sum *S *of all spectral counts obtained from the three replicates was calculated, and the corresponding NSAF_*S *_were deduced and used as a measure of protein abundance. Proteins reproducibly present in all three replicates of at least one of the five species were included in further analyses (1489 proteins). Here we focus on proteome differences between the three fruiting species CH, EX and NZ. Using NSAF_*i*_, we performed Wilcoxon Rank tests to determine species-specific expression (CH vs EX+NZ, EX vs CH+NZ and NZ vs CH+EX) and t-tests to determine up- and down-regulation in all pair wise comparisons of the three species (CH vs EX, EX vs NZ, CH vs NZ). The use of t-tests in the pair wise comparison was possible because once a spectral fraction was added to the spectral count data, the resulting NSAF values were normally distributed. However, NSAF distributions in the three species comparisons were not sufficiently normal to warrant the application of a t-test. Hence the Wilcoxon test has been used instead. P-values were adjusted for multiple testing using the Benjamini-Hochberg correction [29].

### Transcript-protein correlations

Correlation of transcript and protein levels was estimated by correlating abundances of a common set and by intersecting lists of species-specific transcripts and proteins. For the former approach log2 fluorescence intensities for transcripts were determined by a separate channel analysis as implemented in the limma package [33]. Three such analyses with different pre-processing parameters were done with the most conservative approach yielding log2 fluorescence intensities for 9404 genes. This list was intersected with the list of 1489 proteins and their respective NSAF_*S*_, resulting in a common set of 1074 loci. Given the non-normal distributions of transcript and protein data, Spearman-correlation coefficients were calculated to estimate correlations within transcriptomes, within proteomes and within transcriptomes and proteomes of each species. Lists of differentially expressed transcripts and proteins were obtained as described above and intersected to determine the overlap between them (figure 1). Statistical significance of this overlap was evaluated by comparison with a distribution of overlap values generated from 10000 random calculations.

### Metabolite analysis

#### Glucosinolates

For glucosinolate analysis, 100 mg of freeze-dried leave tissue from 8-13 plants of each of the five species was extracted with 80% methanol (v:v) and HPLC-UV-quantified after conversion into desulfoglucosinolates as previously described [3]. Only glucosinolate profiles from the three fruiting species were analyzed. Profiles from individual plants were clustered using Euclidean distance and the Ward clustering algorithm as implemented in the R package cluster [34]. 

#### *Glucosinolate hydrolysis products*

Glucosinolates along with their glucosinolate hydrolysis products were determined from five *P. cheesemanii *(accession: Bob's cove) and *P. novae-zelandiae *(accession: Old man range) plants after autolysis of fresh plant samples in water, extraction of glucosinolate hydrolysis products into dichloromethane and analysis by gas chromatography-flame ionisation detection as described in [3].

## Authors' contributions

CV and PBH undertook plant growth, sampling and RNA extractions. MM, DP and PAH obtained and analyzed protein data. LL, SS and BJ labeled and hybridized RNA samples to microarrays and provided expression raw data. MR quantified glucosinolates and glucosinolate hydrolysis products. CV analyzed transcript, protein and metabolite data. CV and PJL wrote the paper. All authors edited and approved the final manuscript.

## Supplementary Material

Additional file 1 **Table S1**. Table S1 summarises expression statistics for differentially expressed transcripts and proteins. Six worksheets contain details on up-regulated transcripts and proteins in *P. cheesemanii, P. exile *and *P. novae-zelandiae*, respectively. Click here for file

Additional file 2 **Figure S1**. Figure S1 summarises individual and total glucosinolate contents of *P. cheesemanii*, *P. exile *and *P. novae-zelandiae*. Click here for file

Additional file 3 **Table S2**. Table S2 illustrates the microarray hybridization scheme. Click here for file
