# Supplementary material for: Transcript and protein profiling identify candidate gene sets of potential adaptive significance in New Zealand Pachycladon
Source: BMC Evol Biol. 2010 May 20;10:151. doi: 10.1186/1471-2148-10-151 (PMC2886070; doi:10.1186/1471-2148-10-151)
Supplement: Additional file 2 — Figure S1. Figure S1 summarises individual and total glucosinolate contents of P. cheesemanii, P. exile and P. novae-zelandiae. [file 1471-2148-10-151-S2.DOC]

**Figure S1** Mean and standard deviations of fourteen glucosinolates, total glucosinolate contents, C3/C4 glucosinolates combined and methylthio/methylsulfinyl/alkenyl/ glucosinolates combined in *P. cheesemanii* (CH, n=12), *P. exile* (EX, n=13) and *P. novae-zelandiae* (NZ, n=12). Abbreviations: 3MTP, 3-methylthiopropyl glucosinolate; 4MTB, 4-methylthiobutyl glucosinolate; 3MSOP, 3-methylsulfinylpropyl glucosinolate; 4MSOB, 4-methylsulfinylbutyl glucosinolate; Allyl, 2-propenyl glucosinolate; 3-Butenyl, 3-butenyl glucosinolate; S-2OH3-butenyl, *S*-2*-*hydroxy-3*-*butenyl glucosinolate; 6MSOH, 6-methylsulfinylhexyl glucosinolate; 7MTH, 7-methylthioheptyl glucosinolate 7MSOH, 7-methylsulfinylheptyl glucosinolate; 8MSOO, 8-methylsulfinyloctyl glucosinolate; 1MOI3M, 1-methoxy-indolyl-3-methyl glucosinolate; 4OHI3M, 4-hydroxy-indolyl-3-methyl glucosinolate, 4MOI3M, 4-methoxy-indolyl-3-methyl glucosinolate.

Glucosinolate content in µmol/g leaf dry weight

CH EX NZ

CH EX NZ

CH EX NZ

CH EX NZ
