# Supplementary material for: Transcript and protein profiling identify candidate gene sets of potential adaptive significance in New Zealand Pachycladon
Source: BMC Evol Biol. 2010 May 20;10:151. doi: 10.1186/1471-2148-10-151 (PMC2886070; doi:10.1186/1471-2148-10-151)
Supplement: Additional file 3 — Table S2. Table S2 illustrates the microarray hybridization scheme. [file 1471-2148-10-151-S3.DOC]

**Table S2** Hybridization scheme of 36 microarrays.

| Slide | Cy3 | Cy5 | FileName |
| --- | --- | --- | --- |
| loop design - roots (10 arrays) | | | |
| cp1 | CHR | EXR | CP1.gpr |
| cp2 | EXR | CHR | CP2.gpr |
| cp8 | EXR | NZR | CP8.gpr |
| cp9 | NZR | EXR | CP9.gpr |
| cp6 | NZR | FAR | CP6.gpr |
| cp7 | FAR | NZR | CP7.gpr |
| cp10 | FAR | ENR | CP10.gpr |
| cp11 | ENR | FAR | CP11.gpr |
| cp16 | ENR | CHR | CP16.gpr |
| cp17 | CHR | ENR | CP17.gpr |
| loop design - leaves (14 arrays) | | | |
| cp12 | CHL | EXL | CP12.gpr |
| cp13 | EXL | CHL | CP13.gpr |
| cp18 | EXL | NZL | CP18.gpr |
| cp19 | NZL | EXL | CP19.gpr |
| co27 | EXL | NZL | CO27.gpr |
| co26 | NZL | EXL | CO26.gpr |
| cp14 | NZL | FAL | CP14.gpr |
| cp15 | FAL | NZL | CP15a.gpr |
| co29 | NZL | FAL | CO29.gpr |
| co28 | FAL | NZL | CO28.gpr |
| cp3 | FAL | ENL | CP3.gpr |
| cp4 | ENL | FAL | CP4.gpr |
| cp20 | ENL | CHL | CP20.gpr |
| cp21 | CHL | ENL | CP21.gpr |
| loop design - roots and leaves (12 arrays) | | | |
| co25 | CHL | CHR | CO25.gpr |
| co24 | CHR | ENL | CO24.gpr |
| co23 | ENL | FAR | CO23.gpr |
| co22 | FAR | CHL | CO22.gpr |
| co21 | CHL | EXR | CO21.gpr |
| co20 | EXR | ENL | CO20.gpr |
| co19 | ENL | ENR | CO19.gpr |
| co18 | ENR | NZL | CO18.gpr |
| co15 | ENR | NZL | CO15.gpr |
| co17 | NZL | NZR | CO17.gpr |
| co14 | NZL | NZR | CO14.gpr |
| co16 | NZR | CHL | CO16.gpr |

A separate loop was hybridized for roots and leaves and another loop was hybridized to allow for root-leave comparisons. Hybridizations involving leaf samples of *P. novae-zelandiae* used more replicates as there was more variation in the efficiency of dye labelling. Abbreviations: CHL, *P. cheesemanii* leaves; CHR, *P. cheesemanii* roots; FAL, *P. fastigiatum* leaves; FAR, *P. fastigiatum* roots, EXL, *P. exile* leaves; EXR, *P. exile* roots; ENL, *P. enysii* leaves; ENR, *P. enysii* roots; NZL, *P. novae-zelandiae* leaves; NZR, *P. novae-zelandiae* roots.
